# Oridonin induces the apoptosis of metastatic hepatocellular carcinoma cells via a mitochondrial pathway

**DOI:** 10.3892/ol.2013.1541

**Published:** 2013-08-23

**Authors:** MIN ZHU, DUN HONG, YANFANG BAO, CHEN WANG, WEIBO PAN

**Affiliations:** 1 The Public Laboratory, Taizhou Hospital of Zhejiang, Wenzhou Medical College, Linhai, P.R. China; 2 Taizhou Municipal Hospital, Taizhou University Medical School, Taizhou, Zhejiang, P.R. China

**Keywords:** oridonin, MHCC97-H cells, apoptosis, mitochondrial pathway

## Abstract

The selective induction of apoptosis is a promising strategy for cancer therapy. The antitumor effects of oridonin have been reported in several types of malignant tumors. However, the effects of oridonin on MHCC97-H cells, a highly metastatic human hepatocellular carcinoma cell line, have not been reported. The present study aimed to determine the effect of oridonin on the apoptosis of MHCC97-H cells and to identify the underlying molecular mechanisms that are involved. Compared with the untreated control cells, oridonin significantly decreased (P<0.05) cell proliferation in a concentration- and time-dependent manner. Oridonin at concentrations of 12.5, 25, 50 and 100 μM resulted in increased apoptotic Annexin V-positive and propidium iodide-negative cells by 9.5, 15.6, 22.2 and 31.7%, respectively, compared with the control groups (P<0.05). The mitochondrial membrane potential was significantly decreased by 6.0, 12.9, 18.9 and 27.1% in the MHCC97-H cells that were treated with oridonin at concentrations of 12.5, 25, 50 and 100 μM, respectively, for 24 h compared with the control groups (P<0.05). Oridonin increased the activity of caspase-3 and the expression of cleaved caspase-9 and cytochrome c in the cytoplasm and decreased the Bcl-2:Bax ratio in a concentration-dependent manner. The data indicate that oridonin inhibited the proliferation of the MHCC97-H cells by inducing apoptosis via a mitochondrial pathway. This mitochondrial pathway of apoptosis involved a reduction in the mitochondrial membrane potential and the subsequent release of cytochrome c and activation of caspase-3 and -9.

## Introduction

Hepatocellular carcinoma (HCC) is one of the most frequently occurring cancers in the world, resulting in approximately one million deaths every year (1). The majority of liver cancers are diagnosed at later stages due to the absence of symptoms in patients and an incorrect liver disease diagnosis. Surgical options for patients with HCC include a resection of the primary tumor and liver transplantation (2). As HCC is typically diagnosed at an advanced stage, a resection of the primary tumor is typically not an option and >80% of HCC patients have recurrent disease within two years following the surgery. Recurrence and metastasis are the two main causes of patient mortality. Recent advances in our understanding of the biology and signaling pathways of HCC have led to apoptosis induction being considered as a new treatment strategy for HCC (3).

Studies have focused on *Rabdosia rubescens*, which is used as a herbal medicine, due to its antitumor effects and lack of serious side-effects (4,5). Oridonin, a diterpenoid that is isolated from *R. rubescens*, has shown antitumor effects in several malignant tumors, including breast and cervical carcinoma and lymphoma (6,7). Oridonin has been demonstrated to induce apoptosis in HepG2 HCC cells, which have a moderate metastatic potential (8,9). However, the effect of oridonin on human HCC cell lines with a high metastatic potential has not been determined. Therefore, the present study investigated the effect of oridonin on the apoptosis of the highly metastatic MHCC97-H HCC cell line and the underlying molecular mechanism involved.

## Materials and methods

### Reagents

High glucose Dulbecco’s modified Eagle’s medium (DMEM) and fetal calf serum (FCS) were purchased from HyClone (Beijing, China). The Annexin V Alexa Fluor 488/propidium iodide (PI) Apoptosis, MTS/PMS Cell Proliferation Assay, Active Caspase-3 Staining and Cytoplasmic and Mitochondrial Protein Extraction kits were purchased from Invitrogen (Carlsbad, CA, USA), Promega (Madison, WI, USA), Biovision (Milpitas, CA, USA) and Sangon Biotech Co. Ltd (Shanghai, China), respectively. The Z-LEHD-FMK caspase-9 inhibitor, rhodamine-123 and 3,3′-diaminobenzidine tetrahydrochloride (DAB) were purchased from R&D Systems (Minneapolis, MN, USA), Sigma Chemical Co. (St. Louis, MO, USA) and Dako (Glostrup, Denmark), respectively. Cytochrome c, Bcl-2 and Bax monoclonal antibodies and horseradish peroxidase (HRP)-conjugated secondary antibodies (goat-anti-rabbit and goat-anti-mouse) were purchased from Epitomics (Burlingame, CA, USA). Caspase-9 and glyceraldehyde 3-phosphate dehydrogenase monoclonal antibodies were purchased from Cell Signaling (Danvers, MA, USA). Oridonin (Lot, 111721–200501; 97% purity) was obtained from the Beijing Institute of Biological Products (Beijing, China). Oridonin was prepared in dimethyl sulfoxide (DMSO).

### Cell culture

The human MHCC97-H HCC cell line was obtained from the Hepatic Carcinoma Institute, Fudan University (Shanghai, China). The MHCC97-H cells were cultured in DMEM supplemented with 10% FCS at 37°C in a humidified atmosphere, with 5% CO_2_(10). All the experiments were performed with cells in the logarithmic growth phase. The DMSO concentration in the cell cultures (<0.5%) did not affect the cell viability.

### MTS/PMS assay for cell proliferation

The MHCC97-H cells were seeded into 96-well plates at a density of 1×10^5^ cells/ml. The cells were treated with oridonin at concentrations of 6.25, 12.5, 25, 50 and 100 μM for 24, 48 and 72 h. The untreated cells served as the controls. Proliferation was determined using the MTS/PMS Cell Proliferation Assay kit, according to the manufacturer’s instructions. MTS/PMS (10 μl) was added to each well and incubated at 37°C for 2 h. The absorbance was measured at 490 nm on a multi-well plate reader. The effect of oridonin on cell proliferation was reported as the cell survival percentage, calculated as absorbance (oridonin-treated group)/absorbance (control group) × 100. The background absorbance of the medium in the absence of the cells was subtracted from the absorbance values for the control and oridonin-treated groups. Each assay was performed in triplicate and the results are presented as the mean ± SD.

### Annexin V/PI assay for apoptosis

The MHCC97-H cells (1×10^5^ cells/ml) were seeded onto 6-well plates and treated with oridonin at concentrations of 12.5, 25, 50 and 100 μM for 24 h. In addition, the cells were treated with 100 μM oridonin in the presence of 20 μM Z-LEHD-FMK. The apoptotic cells were detected using the Annexin V Alexa Fluor 488/PI Apoptosis kit, according to the manufacturer’s instructions. The cells were washed twice with ice-cold phosphate-buffered saline (PBS), then resuspended in PBS (100 μl) and incubated with Annexin V labeling solution (5 μl) for 30 min at 4°C in the dark. The cells were incubated in 1X buffer solution (200 μl) and labeled with PI. The percentage of the apoptotic cells was determined by flow cytometry (FACScan; Becton Dickinson Corporation, Franklin Lakes, NJ, USA).

### Mitochondrial membrane potential

The MHCC97-H cells were treated with oridonin (12.5, 25, 50 and 100 μM) for 24 h, washed twice with PBS, labeled with rhodamine-123 (1 μg/ml) for 10 min at 37°C and washed twice again with PBS. The mitochondrial membrane potential was determined by flow cytometry.

### Caspase-3 activity

The MHCC97-H cells were treated with oridonin (12.5, 25, 50 and 100 μM) for 24 h, washed twice with PBS and then resuspended in PBS (300 μl). Caspase-3 activity was determined using the Active Caspase-3 Staining kit, according to the manufacturer’s instructions.

### Western blot

Following oridonin treatment, the MHCC97-H cells were washed twice with PBS, lysed with RIPA buffer on ice and centrifuged at 10,000 × g for 30 min at 4°C. The supernatant was collected and stored at −80°C. The cytoplasmic and mitochondrial proteins were extracted using the Cytoplasmic and Mitochondrial Protein Extraction kit, according to the manufacturer’s instructions. The protein concentration was determined using the BCA Protein Assay kit (Sangon Biotech Co. Ltd). Proteins (20 μg) in an equal volume of 2X sample loading buffer were denatured by boiling for 5 min. Electrophoresis was performed at 70 V for 20 min and then at 100 V for 1 h. The proteins were transferred onto polyvinylidene difluoride (PVDF) membranes (0.22 μm) using the semi-dry transfer method (Trans-blot SD; Bio-Rad Laboratories, Hercules, CA, USA). The PVDF membrane was incubated with primary antibody (1:1,000) overnight, washed three times with PBS supplemented with Tween 20 (PBST) for 10 min, incubated with secondary antibody (1:3,000) for 2 h at room temperature and washed twice with PBST for 10 min. Western blot bands were developed using DAB as the HRP substrate and analyzed using Quantity One 4.6 (Bio-Rad).

### Cell morphology

The MHCC97-H cells were treated with 50 μM oridonin at time-points ranging from 24 to 72 h, washed in PBS, dried and stained using a Wright-Giemsa stain. The cell morphology was observed under a light microscope (Leica, Solms, Germany). The untreated cells were used as the controls.

### Statistical analysis

The data are presented as the mean ± SD of three independent experiments. Fisher’s least significant difference (LSD) tests were performed using SPSS version 13.0 software (SPSS, Inc., Chicago, IL, USA). The t-test was applied to compare the means from the two groups. An LSD t-test was utilized to compare the means from multiple groups. The correlation between caspase-3 activity and inducing concentrations of oridonin was analyzed by linear correlation analysis. P<0.05 was considered to indicate a statistically significant difference.

## Results

### Effect of oridonin on the proliferation of MHCC97-H cells

**Figure 1:**
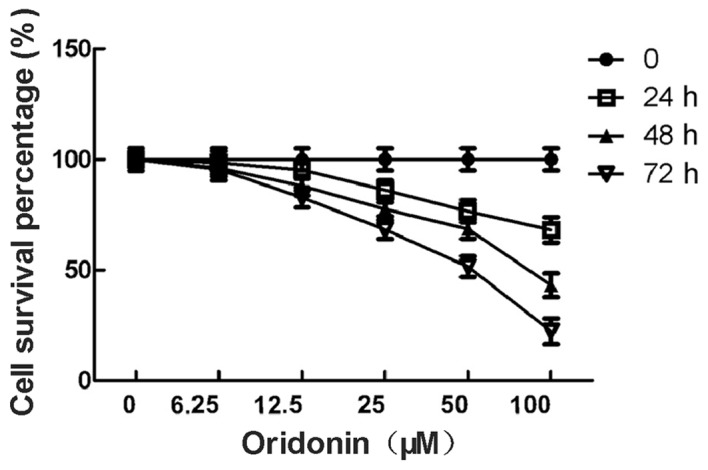
Effect of oridonin on the viability of MHCC97-H cells. The cells were treated with oridonin at concentrations of 0, 6.25, 12.5, 25, 50 and 100 μM for 24–72 h. Cell viability was determined using the MTS/PMS assay and reported as the cell survival percentage. Data are presented as the mean ± SD of three independent experiments.

The percentage of viable MHCC-97-H cells in the oridonin-treated group is shown in Fig. 1. The percentage of viable cells was 98.6, 95.3, 86.2, 76.6 and 68.2% for the cells that were treated with 6.25, 12.5, 25, 50 and 100 μM oridonin for 24 h, respectively. The percentage of viable cells was 96.2, 88.1, 77.5, 68.5 and 43.2% for the cells that were treated with 6.25, 12.5, 25, 50 and 100 μM oridonin for 48 h, respectively. The percentage of viable cells was 95.5, 82.8, 68.3, 51.6 and 22.4% for the cells that were treated with 6.25, 12.5, 25, 50 and 100 μM oridonin for 72 h, respectively. The difference between the test group and the control group was significant (P<0.05). The data indicated that the growth inhibitory effect of oridonin on the MHCC97-H cells was dependent on the concentration and the duration of the treatment. The 6.25 μM concentration of oridonin was not included in the further experiments due to the weak anti-proliferative effect.

### Effect of oridonin on the apoptosis of MHCC97-H cells

**Figure 2:**
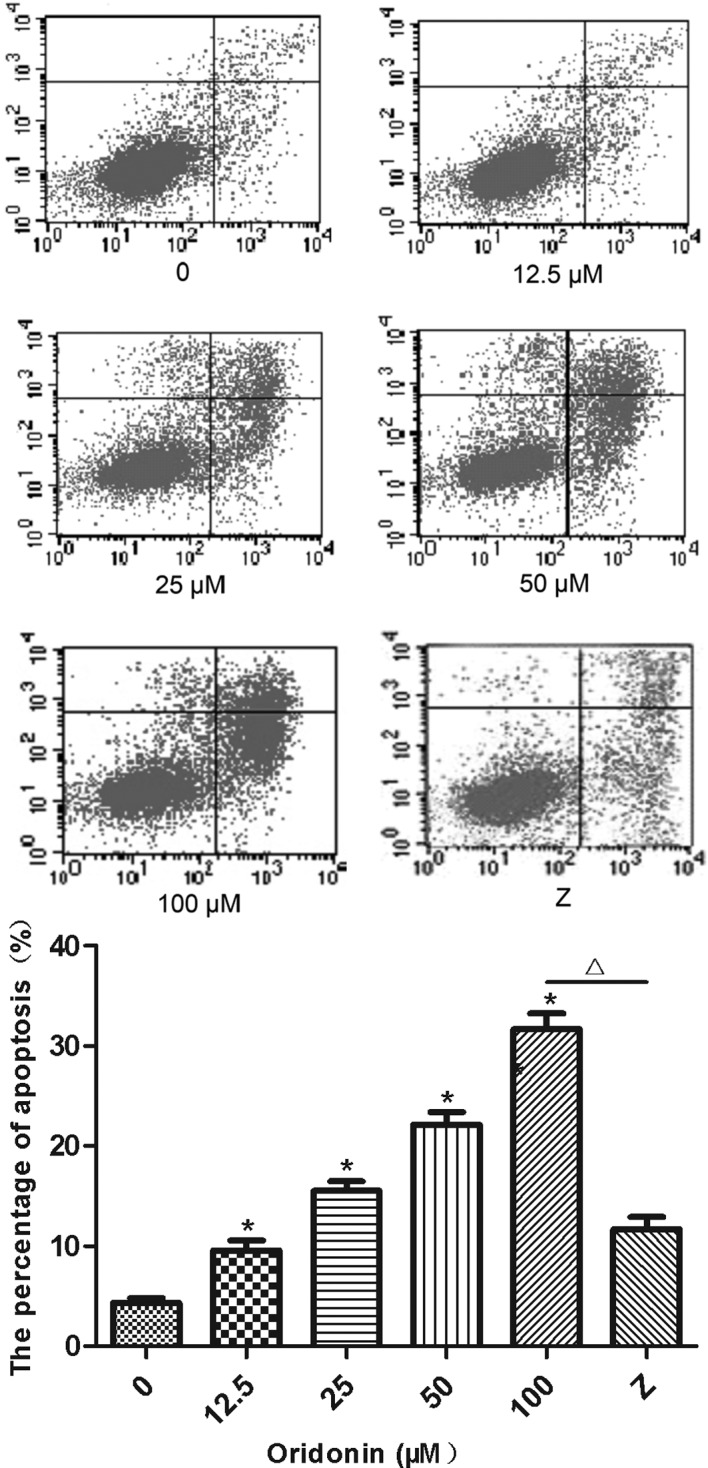
Effect of oridonin on apoptosis in MHCC97-H cells. The cells were treated with the indicated concentrations of oridonin for 24 h. Apoptosis was determined using Annexin V/PI staining. Data are presented as the mean ± SD of three independent experiments. Z represents cells that were treated with oridonin (100 μM) and the caspase-9 inhibitor Z-LEHD-FMK (20 μM).^*^P<0.05 compared with the control cells (LSD t-test). ^Δ^P<0.01 compared with the cells that were treated with 100 μM oridonin alone (t-test). LSD, least significant difference; PI, propidium iodide.

The percentage of Annexin V-positive and PI-negative MHCC97-H cells that were treated with oridonin is shown in Fig. 2. The percentage of the apoptotic cells was significantly higher (P<0.05) for the oridonin-treated cells than for the untreated control cells. Furthermore, oridonin increased the percentage of apoptotic cells in a concentration-dependent manner. The percentage of apoptotic cells (Annexin V-positive and PI-negative) was 9.5, 15.6, 22.2 and 31.7% in the MHCC97-H cells that were treated with 12.5, 25, 50 and 100 μM oridonin for 24 h. Addition of the caspase-9 inhibitor, Z-LEHD-FMK, significantly lowered (P<0.05) the percentage of apoptotic cells that were induced by the 100-μM concentration of oridonin (11.7 vs. 31.7%).

### Effect of oridonin on the mitochondrial membrane potential of MHCC97-H cells

**Figure 3:**
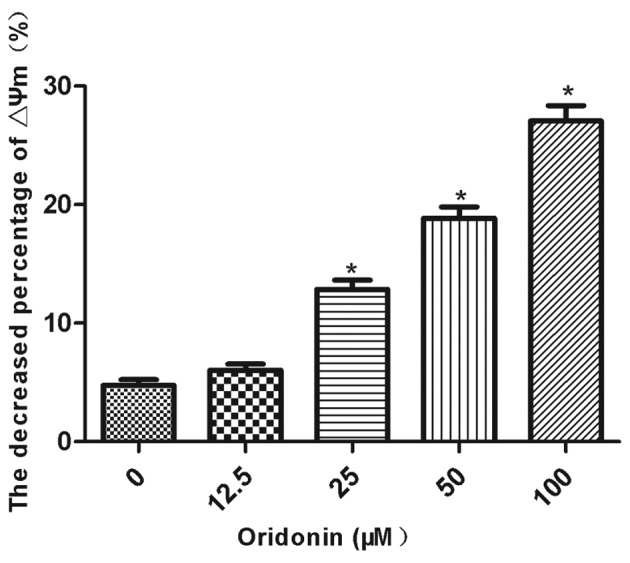
Effect of oridonin on the mitochondrial membrane potential (Δψm) of MHCC97-H cells. The cells were treated with the indicated concentrations of oridonin, and the mitochondrial membrane potential was determined. Data are presented as the mean ± SD of three independent experiments. ^*^P<0.05 compared with the control cells using an LSD t-test. LSD, least significant difference.

The mitochondrial membrane potential was decreased by oridonin in a concentration-dependent manner. The mitochondrial membrane potential was significantly decreased (P<0.05) by 12.9, 18.9 and 27.1% in the MHCC97-H cells that were treated with 25, 50 and 100 μM oridonin, respectively (Fig. 3). By contrast, the decrease in the mitochondrial membrane potential (6.0%) was not significantly different between the 12.5 μM oridonin-treated cells and the control cells. The ratio of the mitochondrial membrane potential prior to and following the oridonin treatment is shown in Fig. 3.

### Effect of oridonin on caspase-3 activity of MHCC97-H cells

**Figure 4:**
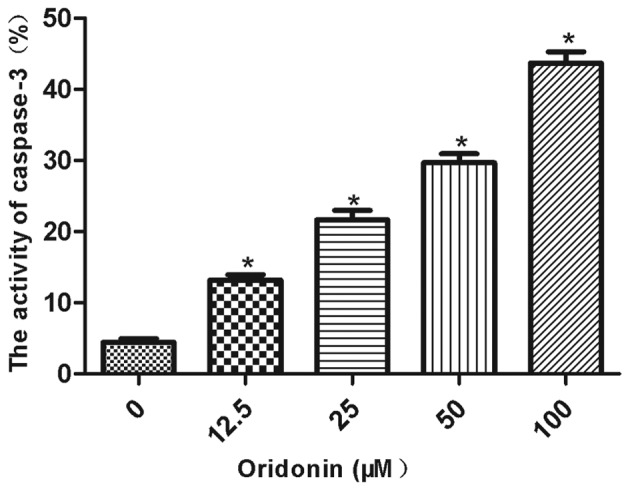
Effect of oridonin on the caspase-3 activity of MHCC97-H cells. The cells were treated with the indicated concentrations of oridonin, and caspase-3 activity was determined. Data are presented as the mean ± SD of three independent experiments. ^*^P<0.05 compared with the control cells (LSD t-test). LSD, least significant difference.

Oridonin increased caspase-3 activity in the MHCC97-H cells (Fig. 4). Caspase-3 activity was significantly increased (P<0.05) by 13.2, 21.6, 29.7 and 43.6% in the cells that were treated with 12.5, 25, 50 and 100 μM oridonin for 24 h. Caspase-3 activity was positively correlated with the oridonin concentration (r^2^=0.9538).

### Effect of oridonin on the expression of apoptotic proteins in MHCC97-H cells

**Figure 5:**
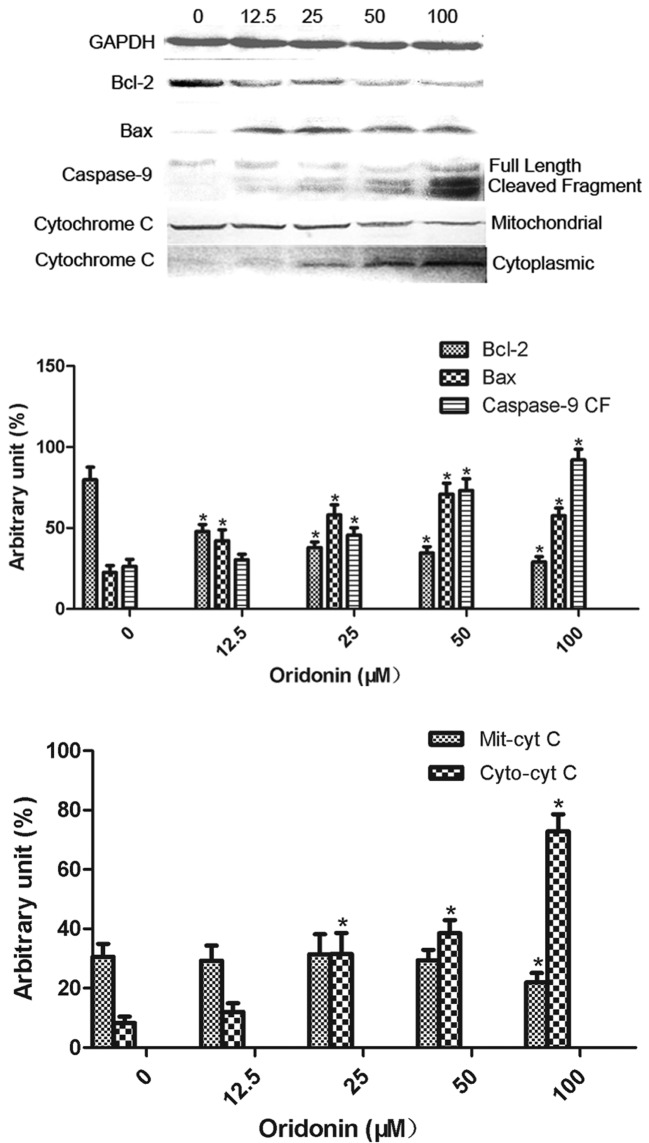
The effect of oridonin on the expression of apoptosis-related proteins in the MHCC97-H cells. The cells were treated with the indicated concentrations of oridonin. The expression of Bcl-2, Bax, caspase-9 and cytochrome c was determined using western blot and normalized to glyceraldehyde 3-phosphate dehydrogenase expression. Data are presented as the mean ± SD of three independent experiments. ^*^P<0.05 compared with the control cells (LSD t-test). CF, cleaved fragment of caspase-9; LSD, least significant difference.

The effect of oridonin at concentrations of 12.5, 25, 50 and 100 μM on the expression of the apoptosis-related proteins, including Bcl-2, Bax, cytochrome c and caspase-9, is shown in Fig. 5. Bcl-2 expression was significantly decreased (P<0.05) and Bax expression was significantly increased (P<0.05) by all the concentrations of oridonin. The expression of cleaved caspase-9 and cytoplasmic cytochrome c was significantly increased (P<0.05) by oridonin at concentrations of 25, 50 and 100 μM. Oridonin at a concentration of 100 μM significantly decreased (P<0.05) the expression of mitochondrial cytochrome c.

### Effect of oridonin on the morphology of MHCC97-H cells

**Figure 6:**
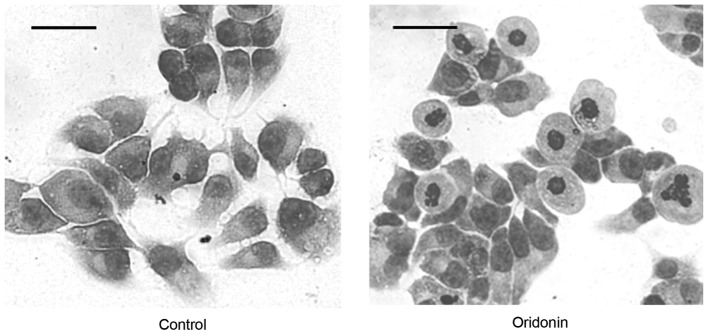
Morphological alterations induced by oridonin in the MHCC97-H cells. The cells were treated with 50 μM oridonin for 24 h and stained with a Wright-Giemsa Stain (magnification, ×200; scale bar, 50 μm).

The untreated control MHCC97-H cells had an epithelioid morphology with a large, round or oval nucleus and abundant cytoplasm (Fig. 6; left panel). The morphological alterations that are associated with cells undergoing apoptosis, including cell shrinkage, nuclear fragmentation and chromatin condensation, were observed in the cells that were treated with 50 μM oridonin for 24 h (Fig. 6; right panel). Necrosis was evident in the cells that were treated with 50 μM oridonin for ≥48 h.

## Discussion

The present study investigated the apoptotic potential of oridonin in MHCC97-H cells, a human hepatoma cell line with a high metastatic capacity (11,12). Oridonin decreased the number of viable cells and increased the percentage of apoptotic cells. Therefore, oridonin inhibited the proliferation of the MHCC97-H cells by inducing apoptosis. The IC_50_ of oridonin, which was calculated using the Bliss method (13), was 142.2, 80.8 and 44.6 μM for the 24-, 48- and 72-h treatments, respectively. These data indicate that the growth inhibitory effect of oridonin is concentration- and time-dependent. Zhang *et al*(14) reported that the MHCC97-H cell line has a higher invasive and metastatic potential than HepG2 and SMMC7721 HCC cell lines. Previously, we reported that the 24 h IC_50_ of oridonin in HepG2 cells was 27.6 μM (8). These findings are in agreement with those of Huang *et al*(9). Taken together, these findings indicate that the effective inhibitory concentration of oridonin is higher in MHCC97-H cells than in HepG2 cells and is likely to be the result of the various metastatic potentials of these cell lines.

The present study indicates that a mitochondrial pathway is involved in oridonin-induced apoptosis in MHCC97-H cells. Cell apoptosis or programmed cell death is significant in the maintenance of the intrinsic stability of multicellular organisms (15). Previous studies have indicated that interactions between multiple genes and their composite regulation were involved in the induction of apoptosis (15–19). It is now known that membrane receptor and mitochondrial pathways are the main regulators of apoptosis. Mitochondrial pathways play significant roles in the regulation of apoptosis by inducing the mitochondrial permeability transition pore (20). Pro-apoptotic factors induce mitochondrial permeability transition pore formation, which leads to the loss of membrane potential and cytochrome c release into the cytoplasm. Cytochrome c binds caspase-9 and Apaf-1 to form a complex that activates other caspase family members, including caspase-3 and caspase-6, to induce apoptosis (21). The present study identified that oridonin decreased the mitochondrial membrane potential and mitochondrial cytochrome c expression and increased cytoplasmic cytochrome c expression in the MHCC97-H cells. Furthermore, the activity of caspase-9 and caspase-3 was increased by oridonin, while the caspase-9 inhibitor, Z-LEHD-FMK, decreased oridonin-induced apoptosis. Taken together, these findings indicate that a mitochondrial pathway is involved in oridonin-induced apoptosis in MHCC97-H cells.

Members of the Bcl-2 family, including Bcl-2 and Bax, play a significant role in the mitochondrial pathway of apoptosis (22,23). Bax is a pro-apoptotic factor that is located in the mitochondrial matrix. Bcl-2 is an anti-apoptotic factor that is located in the outer layer of the mitochondrial membrane. Bax and Bcl-2 regulate apoptosis by controlling the activity of proteases and nucleases. Bax promotes apoptosis in response to certain mitochondrial stimuli by inducing the opening of the mitochondrial permeability transition pore to release cytochrome c. Bcl-2 antagonizes the action of Bax by preventing the opening of the mitochondrial permeability transition pore (23). The present study demonstrated that oridonin decreased Bcl-2 expression and increased Bax expression in a concentration-dependent manner, resulting in a decreased Bcl-2/Bax ratio. The study indicated that oridonin induced apoptosis in the MHCC97-H cells. Therefore, these proteins may be involved in oridonin-induced apoptosis.

In summary, oridonin inhibited the proliferation of the MHCC97-H cells by promoting apoptosis. Oridonin induced apoptosis via a mitochondrial pathway that involved a reduction in the mitochondrial membrane potential to promote the release of cytochrome c and the activation of caspase-3 and -9. Further investigation of the molecular mechanisms by which oridonin induces apoptosis is required.
